# Adherence to physical activity and screen time recommendations of youth: Demographic differences from National Health and Nutrition Examination Survey 2017–2018

**DOI:** 10.1002/osp4.776

**Published:** 2024-07-04

**Authors:** Amin Mokari‐Yamchi, Keith Brazendale, Amir Hossein Faghfouri, Yousef Mohammadpour, Shahsanam Gheibi

**Affiliations:** ^1^ Maternal and Childhood Obesity Research Center Urmia University of Medical Sciences Urmia Iran; ^2^ Department of Health Sciences University of Central Florida Orlando Florida USA; ^3^ Department of Medical Education School of Medicine Urmia University of Medical Sciences Urmia Iran

**Keywords:** NHANES, physical activity, screen time, youth

## Abstract

**Background:**

Engaging in physical activity (PA) and reducing sedentary behaviors among youth are linked to improved mental and physical health. This study aimed to examine demographic differences among youth adhering to PA and Screen Time (ST) recommendations.

**Methods:**

The present study utilized data from the 2017–2018 National Health and Nutrition Examination Survey (NHANES). The NHANES survey employed a cross‐sectional design and gathered information on the daily duration of moderate‐to‐vigorous PA lasting 60 min or more, as well as the maximum daily ST not exceeding 2 h. The analysis encompassed a total of 1697 youth aged between 6 and 17 years.

**Results:**

Overall, 36.3% of participants adhered to PA recommendations, 20.9% adhered to ST recommendations and 10.8% of youth met both recommendations. The odds of meeting PA, ST and both recommendations were inversely associated with obesity (obese vs. normal: aOR, 0.56 [95% CI, 0.42–0.75]), (aOR, 0.67 [95% CI, 0.48–0.94]) and (aOR, 0.51 [95% CI, 0.32–0.82]) respectively, and age (14–17 years vs. 6–9 years: aOR, 0.2 [95% CI, 0.15–0.27]), (aOR, 0.33 [95% CI, 0.23–0.47]) and (aOR, 0.16 [95% CI, 0.09–0.3]) respectively.

**Conclusion:**

A small portion of the youth met PA and ST recommendations. Older youth, youth with obesity, and youth with a parent or guardian who had not completed a high school education were particularly at risk.

## 1 INTRODUCTION

Physical activity (PA) and sedentary behaviors significantly impact the overall health and well‐being of youth, as emphasized by the World Health Organization and the American Academy of Pediatrics.^1^, ^2^ While regular PA is crucial for healthy development and weight management,^3^ excessive screen time (ST) has been linked to various negative health outcomes, including obesity, poor sleep quality, behavioral issues, and academic performance.^4^ Adhering to PA recommendations of at least 60 min of moderate‐to‐vigorous activity per day, as well as limiting ST viewing to no more than 1 h per day, has been strongly recommended due to its positive impact on the health and well‐being of young individuals.^5^

The PA and ST levels among youth have also been found to differ based on socioeconomic status (SES).^6^, ^7^, ^8^ However, the findings have not been consistent. For example, the National Youth Fitness Survey survey showed that 3–15 year old youth from less affluent families had lower levels of PA participation.^9^ On the other hand, a national study conducted in Vietnam^10^ and a survey conducted in rural area of United States (US)^11^ demonstrated that individuals from low SES backgrounds tended to be more physically active compared to those from higher SES backgrounds. It has also been observed that children's ST patterns differ based on their family's SES. Salway et al. found that youth living in households with lower levels of SES had higher levels of ST.^8^ However, Mollborn et al. children from families with college‐educated parents devoted an average of 16 h each week to non‐TV‐related technology usage, which was 6 h greater than those from the lowest SES group.^12^ Recognizing the disparities in PA and ST among specific sociodemographic groups is vital for public health initiatives, as these findings are commonly used to identify target populations for interventions aimed at increasing PA levels and managing ST viewing among youth.

Due to inconsistent findings and limited knowledge surrounding youth's adherence to recommendations for PA and ST, this study aimed to address this gap by investigating the relationships between demographic factors and PA and ST behaviors among youth in the US. To achieve this goal, the National Health and Nutrition Examination Survey (NHANES) data were used.

## 2 METHODS

### 2.1 Study design

The 2017–2018 NHANES provided the data for this study. NHANES is an ongoing survey that aims to assess the health and nutritional status of both adults and children residing in the US. NHANES uses a multi‐stage probability sampling technique to select its participants. The population studied in the present work consisted of 1697 youth aged 6–17 years. Participants aged 12 and over were interviewed directly. For survey participants under 12, a proxy respondent, and most often a parent answered the interview questions.

### 2.2 Measures

The analysis incorporated demographic factors such as age, gender, ethnicity, and three indicators of SES: family income to poverty recommendations ratio (FIPR), highest educational attainment of the household head, and marital status of the household head. The head of household was defined as the person who rents or owns the residence where members of the household live. FIPR was categorized based on the US Department of Health and Human Services' poverty guidelines to determine poverty levels. Low‐income was defined as FIPR less than 1.3, middle‐income as FIPR between 1.3 and 3.5, and high‐income as FIPR greater than 3.5.^13^ Obesity status was defined based on age‐ and sex‐specific BMI percentiles (calculated as weight in kilograms divided by height in meters squared) using the 2000 Centers for Disease Control and Prevention (CDC) growth charts. The study used the CDC percentiles to determine weight status, defining a BMI ≥85th and <95th percentile of the reference population as overweight and ≥95th percentile as obese.^14^ Food security was evaluated using the US Household Food Security Survey Module. A responsible adult answered a total of 10 questions about the household's food security, and an additional eight questions were specifically asked for households containing youth aged 17 or younger (18 questions total). The children's experiences were divided into four distinct levels of food security: high, marginal, low, and very low. In order to assess food security, the high and marginal categories were grouped together, while the low and very low categories were merged to determine food insecurity.

To assess PA, participants were asked a single question about the number of days they engaged in PA for at least 60 min in the past week. Youth who reported being physically active for 7 days per week were considered to meet the recommendations. The validity of this tool has been verified for evaluating whether youth achieve the recommended level of PA.^15^ The study examined ST by posing two inquiries that urged participants to recollect their average daily sitting time for watching TV or videos over the past 30 days, as well as their average daily usage of computers or engagement in computer games outside of work or school during the same time frame. These questions are similar to those utilized in other reputable surveys such as the CDC's Youth Risk Behaviors Surveillance System.^16^, ^17^ To determine whether youth adhered to the recommendations for ST, the time reported in both questions was summed up. If the combined ST was 2 h or less per day, they were classified as meeting the ST recommendations.

### 2.3 Statistical analysis

All analyses were performed using the Statistical Package for Social Sciences, version 22, and *p*‐values < 0.05 were considered statistically significant. A variety of demographic predictors were explored in relation to meeting PA and ST recommendations, including age, gender, race/ethnicity, FIPR, obesity status, marital status, household education level, and food security. Multiple logistic regression models were used to identify significant predictors of PA and ST among youth. In this study, adjusted and unadjusted odds ratio (OR) with 95% confidence intervals were reported to examine differences in adherence to recommendations by demographic predictors.

## 3 RESULTS

The study population consisted of 1697 youth aged 6–17 years. Table 1 presents sample sizes and the prevalence of different demographic predictors of meeting PA and ST recommendations. Among the study participants, 616 (36.3%) of youth adhered to PA recommendations, 355 (20.9%) adhered to ST recommendations, and 183 (10.8%) youth met both PA and ST recommendations. Table 1 shows the associations between adherence and PA and ST recommendations by demographic characteristics and obesity status (Table 1).

**TABLE 1:** Characteristics of children who met the Physical Activity or Screen‐Time recommendations from 2017 to 2018 NHANES.

Descriptive characteristic	Sample size	Met physical activity recommendation^a^	Met screen‐time recommendation^b^	Met both recommendations
	*N*	*N* (%)	*N* (%)	*N* (%)
Overall	1697	616 (36.3)	355 (20.9)	183 (10.8)
Gender				
Male	857	326 (38)	184 (21.5)	97 (11.3)
Female	840	290 (34.5)	171 (20.4)	86 (10.2)
Age group, y				
6–9	639	343 (53.7)	176 (27.5)	113(17.7)
10–13	576	185 (32.1)	123 (21.4)	54 (9.4)
14–17	482	88 (18.3)	56 (11.6)	16 (3.3)
Race/ethnicity				
Hispanic	419	122 (29.1)	94 (22.4)	39 (9.3)
Non–Hispanic White	523	208 (39.8)	143 (27.3)	81(15.5)
Non–Hispanic Black	410	173 (42.2)	57 (13.9)	35 (8.5)
Other races and multiracial	345	113 (32.8)	61 (17.7)	28 (8.1)
Obesity status				
Normal	974	389 (39.9)	213 (21.9)	124 (12.7)
Over weight	294	104 (35.4)	72 (24.5)	28 (9.5)
Obese	429	123 (28.7)	70 (16.3)	31 (7.2)
Head‐of‐household gender				
Male	735	262 (35.6)	144 (19.6)	71 (9.7)
Female	962	354 (36.8)	211 (21.9)	112 (11.6)
Head‐of‐household education level				
<High school	300	91 (30.3)	60 (20)	26 (8.7)
High school	946	357 (37.7)	189 (20)	96 (10.1)
College graduate ≤	383	145 (37.9)	96 (25.1)	56 (14.6)
Head‐of‐household marital status				
Married	1181	431 (36.5)	264 (22.4)	138 (11.7)
Single	487	179 (36.8)	88 (18.1)	44 (9)
Income (%FIPR)				
<130%	584	225 (38.5)	114 (19.5)	63 (10.8)
130%–349%	614	209 (34)	119 (19.4)	58 (9.4)
≥350%	340	107 (31.5)	93 (27.4)	44 (12.9)
Food security status				
Food secure	1432	525 (36.7)	300 (20.9)	156 (10.9)
Food insecure	223	73 (32.7)	50 (22.4)	23 (10.3)

Abbreviations: FIPR, family income to poverty level ratio; NHANES, National Health and Nutrition Examination Survey.

^a^ Recommendation for physical activity: 60 min/day or more moderate‐to‐vigorous physical activity, 7 d/wk.

^b^ Recommendations for screen‐time viewing: ≤2 h/day.

Table 2 presents the results from the logistic regression analyses. The chances of meeting PA recommendations were notably reduced for adolescents aged 10–13 and 14–17, in comparison to children aged 6–9 (adjusted odds ratio [aOR], 0.47 [95% CI, 0.36–0.61] vs. aOR 0.2 [0.15–0.27]). Youth with obesity were less likely to meet the recommendations for PA, ST, and both compared to those with a normal BMI (aOR: 0.56 [95% CI: 0.42–0.75], 0.67 [95% CI: 0.48–0.94], and 0.51 [95% CI: 0.32–0.82]) respectively. Additionally, youth living in households with an income between 130% and 349% of the poverty line had lower odds of meeting PA recommendations (aOR: 0.69 [95% CI: 0.52–0.92]) compared to those living in households below 130% of the poverty line. Youth in households with an income of 350% or more of the poverty line also had lower odds of meeting PA recommendations (aOR: 0.51 [95% CI: 0.35–0.75]) compared to those living in households below 130% of the poverty line. Non‐Hispanic youth and those who were from households with an adult with higher education were more likely to participate in PA.

**TABLE 2:** Logistic regression analysis of meeting physical activity or screen time recommendations.

		Physical activity^a^	Screen‐time viewing^b^	Both
Variable		Odds ratio (OR)		
Gender				
Model^1^	Male	1	1	1
	Female	0.85 (0.7–1.04)	0.93 (0.74–1.18)	0.89 (0.65–1.21)
Model^2^	Male	1	1	1
	Female	0.82 (0.65–1.03)	1.02 (0.79–1.32)	0.96 (0.69–1.35)
Age group, y				
Model^1^	6–9	1	1	1
	10–13	0.4 (0.32–0.51)	0.48 (0.34–0.68)	0.33 (0.18–0.58)
	14–17	0.19 (0.14–0.25)	0.34 (0.24–0.48)	0.16 (0.09–0.27)
Model^2^	6–9	1	1	1
	10–13	0.47 (0.36–0.61)	0.69 (0.51–0.92)	0.53 (0.36–0.77)
	14–17	0.2 (0.15–0.27)	0.33 (0.23–0.47)	0.16 (0.09–0.3)
Race/ethnicity				
Model^1^	Hispanic	1	1	1
	Non–Hispanic White	1.6 (1.22–2.11)	1.3 (0.96–1.75)	1.78 (1.19–2.67)
	Non–Hispanic Black	1.77 (1.33–2.37)	0.55 (0.38–0.8)	0.9 (0.56–1.46)
	Other races and multiracial	1.18 (0.87–1.61)	0.74 (0.51–1.06)	0.86 (0.51–1.43)
Model^2^	Hispanic	1	1	1
	Non–Hispanic White	1.48 (1.06–2.05)	1.23 (0.87–1.75)	1.48 (0.92–2.37)
	Non–Hispanic Black	1.74 (1.22–2.47)	0.53 (0.35–0.82)	0.75 (0.42–1.33)
	Other races and multiracial	1.06 (0.73–1.55)	0.76 (0.50–1.16)	0.74 (0.41–1.32)
Obesity status				
Model^1^	Normal	1	1	1
	Over weight	0.82 (62–1.08)	1.15 (0.85–1.57)	0.72 (0.46–1.11)
	Obese	0.6 (0.47–0.77)	0.69 (0.51–0.93)	0.53 (0.35–0.8)
Model^2^	Normal	1	1	1
	Over weight	0.74 (55–1.02)	1.09 (0.78–1.53)	0.59 (0.36–0.96)
	Obese	0.56 (0.42–0.75)	0.67 (0.48–0.94)	0.51 (0.32–0.82)
Head‐of‐household gender				
Model^1^	Male	1	1	1
	Female	1.05 (0.86–1.28)	1.15 (0.9–1.46)	1.23 (0.9–1.68)
Model^2^	Male	1	1	1
	Female	1.08 (0.84–1.4)	1.22 (0.92–1.63)	1.35 (0.93–1.96)
Head‐of‐household education level				
Model^1^	<High school	1	1	1
	High school	1.39 (1.05–1.84)	1 (0.72–1.38)	1.19 (0.75–1.87)
	College graduate ≤	1.4 (1.01–1.93)	1.33 (0.92–1.92)	1.8 (1.1–2.95)
Model^2^	<High school	1	1	1
	High school	1.43 (1.02–2)	1.27 (0.87–1.87)	1.64 (0.95–2.83)
	College graduate ≤	1.68 (1.09–2.58)	1.51 (0.93–2.44)	2.67 (1.39–5.13)
Head‐of‐household marital status				
Model^1^	Married	1	1	1
	Single	1.01 (0.81–1.26)	0.76 (0.58–1)	0.75 (0.52–1.07)
Model^2^	Married	1	1	1
	Single	0.97 (0.72–1.29)	0.79 (0.57–1.11)	0.72 (0.46–1.12)
Income (%FIPR)				
Model^1^	<130%	1	1	1
	130%–349%	0.82 (0.65–1.04)	0.99 (0.74–1.32)	0.86 (0.59–1.25)
	≥350%	0.73 (0.55–0.97)	1.55 (1.13–2.12)	1.22 (0.81–1.85)
Model^2^	<130%	1	1	1
	130%–349%	0.69 (0.52–0.92)	0.92 (0.67–1.28)	0.68 (0.44–1.03)
	≥350%	0.51 (0.35–0.75)	1.32 (0.87–1.99)	0.77 (0.45–1.32)
Food security status				
Model^1^	Food secure	1	1	1
	Food insecure	0.84 (0.62–1.13)	1.09 (0.77–1.53)	0.94 (0.59–1.49)
Model^2^	Food secure	1	1	1
	Food insecure	0.76 (0.53–1.09)	1.15 (0.78–1.7)	0.93 (0.55–1.59)

*Note*: Model^1^: crude mode. Model^2^: adjusted for all variables shown in the table.

^a^ Recommendation for physical activity: 60 min/day or more moderate‐to‐vigorous physical activity, 7 d/wk.

^b^ Recommendations for screen‐time viewing: ≤2 h/day.

In a similar manner to PA, youth between the ages of 10–13 and 14–17 exhibited a decreased likelihood of adhering to ST recommendations compared with those aged 6–9 (aOR, 0.69 [95% CI [CI], 0.51–0.92]) and (aOR, 0.33 [95% CI, 0.23–0.47]), respectively. Additionally, non‐Hispanic black youth were less likely (aOR, 0.53 [95% CI, 0.35–0.82]) than Hispanic youth to meet the ST recommendations.

The odds of meeting both PA and ST recommendations differed by age (10–13 and 14–17 years (aOR, 0.53 [95% CI, 0.36–0.77]) and (aOR, 0.16 [95% CI, [0.09–0.3]) compared to 6–9 years children, obesity status (overweight and obese youth (aOR, 0.59 [95% CI, 0.36–0.96]) and (aOR, 0.51 [95% CI, 0.32–0.82]) compared to normal weight youth and educational level of household head (college degree compare to less than high school (aOR, 1.8 [95% CI, 1.1–2.95]).

## 4 DISCUSSION

In this study, a small portion of the youth were able to meet PA, ST, and both recommendations. However, it was discovered that youth with obesity, Hispanic youth, and older‐aged youth were at greater risk of not meeting recommendations compared with their counterparts. Considering the previous report of the adherence rate for PA and ST among youth based on the 2009–2010 NHANES data^18^ indicates a significant reduction. A similar trend was reported in a longitudinal study where the authors reported during 2011–2019, a notable decrease in the proportion of high school students who successfully met the recommended PA guideline of at least 60 min per day, for all 7 days of the week.^19^

Our findings showed that age was a significant predictor of adherence to PA and ST recommendations among youth, with younger individuals more likely to meet the PA and ST recommendations compared to their older counterparts. These findings are consistent with previous studies that have suggested lower PA and higher ST in older youth.^18^, ^20^, ^21^ For example, using NHANES 2009–2010 data, Fakhouri et al. showed that the proportion of youth meeting PA recommendations decreased from 76.1% of youth aged 6–8 years to 64.7% of youth aged 9–11 years.^18^ This could be due to various factors such as increased academic and social demands as youth get older or more autonomy over how older youth spend their free time, thus leading to decreased PA and increased ST. In addition, older youth potentially have more access to electronic devices and social media, which can contribute to increased ST.^22^

Our results indicate that non‐Hispanic youth were more likely to meet PA recommendations than Hispanic youth, but this difference was not observed in ST recommendations. Data from the Behavioral Risk Factor Surveillance System also showed similar trends in the prevalence of physical inactivity across different ethnic groups. Specifically, Hispanic youth had the highest rate of physical inactivity at 31.7%, followed by non‐Hispanic blacks at 30.3%, and non‐Hispanic whites at 23.4%.^23^ These results suggest that ST viewing and PA level may be separate constructs and that low levels of ST viewing do not necessarily predict higher levels of PA.

This study revealed that youth with obesity had lower odds of meeting PA and ST recommendations. This aligns with previous findings from Anderson et al.^24^ and Fakhouri et al.,^18^ using data from 2001 to 2004 and 2009–2010 NHANES surveys, respectively. These studies indicated that children with obesity were more inclined to have insufficient PA levels, exceed 2 h of ST daily, and engage in both behaviors concurrently compared with children without obesity. This suggests that youth with obesity may be more likely to engage in sedentary behaviors and less likely to meet PA and ST recommendations. This could be due to a variety of factors, such as physical limitations, lack of motivation or interest in PA, or societal stigma and discrimination that may discourage youth with obesity from participating in PA.

Another notable finding from our study indicates that youth from households with higher income levels and whose head of household have higher levels of education tend to have higher levels of PA. Possibly, this occurs because parents possessing higher levels of education possess a greater understanding of the health consequences associated with PA and are more inclined to motivate their children to engage in such activities.^25^, ^26^ Ke et al. revealed that parental education appears to be the strongest and most consistent predictor of PA in Chinese youth.^6^ Additionally, family income has been proposed as a determinant of youth's PA levels due to its potential impact on the availability of resources and opportunities for PA. However, the literature has presented conflicting findings in this regard.^11^, ^27^, ^28^ Some studies report that children from low‐income families have low levels of PA, and spend more time sedentary.^28^ Other studies have found that income disparities did not determine children's PA or sedentary behavior.^27^ Additionally, a study by Cottrell and colleagues revealed that children from low‐income households in rural areas were actually more physically active than those from higher income brackets. Moreover, parents from low‐income families were found to be more supportive of their child's PA by encouraging outdoor play, providing positive reinforcement, and participating in PA with their youth.^11^

The study extends the body of evidence on PA and ST research by presenting data from a representative sample of youth in the USA and explores the impact of different sociodemographic indicators on meeting PA and ST recommendations among youth. However, limitations of the study should be recognized. Firstly, utilization of a cross‐sectional design in this study does not provide conclusive evidence of causality. To enhance our comprehension of the connection between sociodemographic factors, PA and ST in youth, it is imperative to conduct longitudinal studies. Secondly, PA was not measured objectively. Self‐reported estimates of PA and ST may be biased and are open to misreporting. Additionally, a limited single‐item question was employed for assessing PA and ST behaviors among youth; thus, the accuracy of the results should be interpreted with caution. To address these measurement issues, it is recommended that future studies utilize objective measures such as accelerometers to accurately assess PA.

In conclusion, adherence to PA and ST recommendations among youth is low, and it appears that many youth reduce their PA and increase their ST as they get older. Age, obesity status, household income, and educational level of the household head were found to be significant predictors of adherence to these recommendations. These findings suggest the need for targeted interventions aimed at increasing PA and reducing ST among youth, particularly those who are older, obese, and from low‐income households. By identifying some of these pertinent factors, public health professionals and policy makers can develop and implement interventions to support those individuals most at risk of not meeting PA and ST recommendations and promote healthy behaviors and environments to improve the overall health and well‐being of youth. Future research, particularly from longitudinal studies, could offer deeper insights into the relationship between PA, ST viewing, and obesity status among youth.

## CONFLICT OF INTEREST STATEMENT

The authors declare that the research was conducted in the absence of any commercial or financial relationships that could be construed as a potential conflict of interest.

## INFORMED CONSENT STATEMENT

The NHANES protocol was reviewed and approved by the National Center for Health Statistics research ethics review board. All participants provided written informed consent.

## Data Availability

Data can be downloaded from the “NHANES” database (https://wwwn.cdc.gov/nchs/nhanes/continuousnhanes/default.aspx?BeginYear=2017, accessed on 30 October 2023).
